# Xp21 DNA microdeletion syndrome in a Chinese family: clinical features show retinitis pigmentosa and chronic granuloma

**DOI:** 10.3389/fgene.2023.1276227

**Published:** 2024-01-26

**Authors:** Mengyang Li, Xueqin Hu, Xueli Wu, Na Zhao, Yuanyuan Lian, Meijiao Ma, Huiping Li, Xunlun Sheng

**Affiliations:** ^1^ Gansu Aier Ophthalmology and Optometry Hospital, Lanzhou, China; ^2^ Ningxia Eye Hospital, People’s Hospital of Ningxia Hui Autonomous Region, Third Clinical Medical College of Ningxia Medical University, Yinchuan, China

**Keywords:** RP GTPase regulator, cytochrome b-245 beta chain, X-linked Kx blood group antigen, retinitis pigmentosa, chronic granulomatous disease, McLeod syndrome, X-linked recessive inheritance

## Abstract

Xp21 DNA microdeletion syndrome is a very rare disease characterized by retinitis pigmentosa (RP), chronic granulomatous disease (CGD), and McLeod syndrome (MLS). Due to the complex and diverse clinical manifestations, early diagnosis remains a challenge for many physicians. In this study, for the purpose of determining the pathogenic gene variants and definitive diagnosis in a patient medically backgrounded with RP and CGD from a normal Chinese family, whole-exome sequencing (WES) was performed in this proband and copy number variation (CNV) was further verified in other family members by qPCR. A genetic evaluation revealed that the short arm of the X chromosome in the proband had a deletion CNV Xp21.1p11.4 (37431123–38186681) of approximately 0.755 Mb in size, and contained three contiguous OMIM genes as X-linked Kx blood group antigen (*XK*), cytochrome b-245 beta chain (*CYBB*), and RP GTPase regulator (*RPGR*). The qPCR results confirmed the copy number loss in Xp21.1p11.4 present in the proband and his unaffected mother. According to the American College of Medical Genetics and Genomics (ACMG) guidelines for the CNV interpretation, the deletion of this segment was a pathogenic variant. Our results provided evidence that CNV deletion of Xp21.1p11.4 in the short arm of the X chromosome was a pathogenic variant in such Chinese RP and CGD family, and the McLeod phenotype was not yet available. This study suggests that genetic testing is essential for a definitive diagnosis, which should better assist physicians in prediction, diagnosis, genetic counseling, and guidance for Xp21 DNA microdeletion syndrome.

## Introduction

Xp21 DNA microdeletion syndrome is a very rare disease characterized by retinitis pigmentosa (RP), chronic granulomatous disease (CGD), and McLeod syndrome (MLS) (Francke et al., 1985; Wilcox et al., 1986; de Saint-Basile et al., 1988; Watkins et al., 2011). Retinitis pigmentosa (RP; MIM: 26800) is the most common inherited retinal dystrophy (IRD), with a global prevalence of approximately 1 in 3,500–5,000 (Anasagasti et al., 2012). Depending on the mode of inheritance, autosomal dominant inheritance accounts for 5%–20%, autosomal recessive inheritance for 15%–20%, and X-linked inheritance for 5%–15% (Tsang and Sharma, 2018a). In addition, the remaining 40%–50% have a specific clinical phenotype with a two-gene RP and a rare mitochondrial inherited RP (Liu et al., 2022). X-linked recessive retinitis pigmentosa (XLRP) is considered one of the most severe forms of RP, accounting for approximately 6%–20% of all RP cases (Tsang and Sharma, 2018b), and 70% of XLRP is due to variants in the *RPGR* gene (Shu et al., 2012). The *RPGR* gene is located in the Xp21.1 chromosomal region and spans 172 kilobases (Patnaik et al., 2015). The protein encoded by *RPGR*, which is located in the connecting cilium (CC) that links the inner and outer segments of photoreceptor cells, facilitates a high metabolic rate of photoreceptors by transporting proteins between photoreceptor cells in the ciliary compartment via intraflagellar transport (IFT) (Hosch et al., 2011). Therefore, any variant in the *RPGR* gene could lead to severe photoreceptor dysfunction, likely inducing apoptosis and ultimately causing retinal degeneration.

The most common *RPGR* gene variants (approximately 54%) are small deletions (1–28 bp) that result in a reading frame shift in the genetic code, followed by nonsense variants (approximately 18%) and missense variants (10%) (Shu et al., 2008). When deletions occur in large segments of the *RPGR* gene on the short arm of the X chromosome Xp21.1, expansion of the variant into the genes surrounding the *RPGR* can result in an “Xp21 DNA microdeletion syndrome” (Arai et al., 2011). The clinical phenotype of these patients is always complex, and special attention to all aspects of the syndrome is required in the treatment.

Chronic granulomatous disease (CGD) is a primary immunodeficiency disease caused by defects in any of the five subunits of the NADPH oxidase complex (gp91^phox^, p47^phox^, p22^phox^, p67^phox^, and p40^phox^) (Arnold and Heimall, 2017). It impairs the function of the NADPH oxidase complex enzymes in neutrophils and monocytes so that the bactericidal capacity of phagocytes is diminished or lost, leading to recurrent infections, dysregulation of the inflammatory response, and consequent granuloma formation (Köker et al., 2013). The condition is characterized by recurrent life-threatening bacterial and fungal infections of the skin, airways, lymph nodes, liver, brain, and bones, and the most common pathogens include *Staphylococcus aureus*, *Aspergillus* species, *Klebsiella* species, *Burkholderia cepacia*, *Serratia marcescens*, and *Salmonella* species (Roos, 2016).

X-linked recessive CGD (X-CGD) accounts for approximately 70% of CGD cases (Jirapongsananuruk et al., 2002) due to a variant in the *CYBB* gene that results in an abnormal gp91^phox^ protein in the encoded NADPH oxidase complex (Köker et al., 2013). Autosomal recessive CGD accounts for approximately 30% of all CGD cases (Justiz-Vaillant et al., 2023), and clinical X-CGD is predominantly a male childhood disease (Lent-Schochet and Jialal, 2023). However, there are also a few reports of diagnosed X-CGD adult female subjects with CGD due to extreme unilateral inactivation of the X chromosome, whose clinical features are similar to those of classical X-CDG patients (López-Hernández et al., 2019). The *CYBB* gene, 30 kb in full length, is located on the short arm of the X chromosome Xp21.1 (Jirapongsananuruk et al., 2002). Deletions and frameshifts, missense, nonsense, or splice site variants of the *CYBB* gene can result in CGD defects and, in some cases, deletions of large segments of the X chromosome, leading to “Xp21 DNA microdeletion syndrome” (Peng et al., 2007). McLeod syndrome (MLS) is a rare progressive disease, an X-linked multisystem disorder encoded by the *XK* gene, with central nervous system, neuromuscular, cardiovascular, and hematological clinical manifestations that may include progressive chorea-like movements, cardiomyopathy, peripheral neuropathy, and cognitive decline accompanied by comorbidities such as psychosis (Wilcox et al., 1986). As previously mentioned, the “Xp21 DNA microdeletion syndrome” is known to link X-linked chronic granulomatous disease to XLRP, McLeod syndrome, and Duchenne muscular dystrophy, among other disorders.

The phenotypic heterogeneity of RP varies significantly among individuals (Bruninx and Lepièce, 2020). XLRP also includes progressive and quiescent disease in addition to symptomatic and asymptomatic disease (De Silva et al., 2021). Approximately 65% of RP is non-syndromic with isolated retinal lesions, and more than 80 genetic variants are known to cause non-syndromic RP. More than 70 genes cause RP syndrome, which involves multiple systems and manifests with complex and diverse pathogenic conditions. Moreover, a single diagnostic criterion is not yet available (Fahim et al., 2020). Therefore, the diagnosis of RP should be based not only on clinical manifestations and specialist examinations but also on genetic testing to identify the pathogenic genes (Fahim, 2018). The advent of whole-exome sequencing technology has improved the level of accurate diagnosis of these diseases and changed the process of defining the phenotypic spectrum of the disease. To date, no cases of both RP and CGD patients have been reported in China, and the pathophysiological link between these two diseases is still unclear. In this study, whole-exome sequencing was used to detect genetic variants in a child with such two rare diseases as RP and CGD, and subsequent analysis of the genotypic and clinical phenotypic characteristics of ocular diseases associated with RP syndrome was made in conjunction with previous reports, all of which will contribute to clinicians further and better understanding these diseases accordingly.

## Materials and methods

### Subjects and clinical data collection

This study complied with the Declaration of Helsinki and was approved and reviewed by the Ethics Committee on Human Research of Gansu Aier Ophthalmology and Optometry Hospital [Approval No. GSAIER2023IRB03]. Informed consent was obtained from the patient and his family for all genetic testing and diagnostic work. The proband (II-1) is a 7-year-old boy born to a healthy unrelated couple (I-1, I-2). All individuals in this family were recruited for both clinical and genetic testing. Comprehensive ophthalmic examinations were performed, including best corrected visual acuity (BCVA), color fundus photography, optical coherence tomography, full-field electroretinography (ERG), and relevant blood laboratory tests.

### Genetic testing

The genomic DNA of the subjects was extracted from peripheral blood. Target gene exons and adjacent splice regions (approximately 20 bp), in addition to the full length of the mitochondrial genome, were captured by probe hybridization and enriched. The enriched genes were subjected to quality control and sequenced using a high-throughput sequencer. BWA software was used to compare the reads to the hg38 human genome reference sequence provided by UCSC. The SNV and InDel variants were identified by GATK’s HaplotypeCaller and then further annotated and screened using the population variant frequency database gnomAD and disease databases ClinVar, Decipher, etc., and bioinformatics prediction software SIFT, Polyphen2, and LRT for further annotation and screening. CNV analysis of the probe coverage region was performed using the xhmm and clamms algorithms.

### Pathogenicity analysis of genetic variants

#### Bioinformatic analysis

For CNV interpretation rules, we refer to the 2019 edition of the American College of Medical Genetics and Genomics (ACMG) Guidelines for Interpretation and Reporting of Copy Number Variation (CNV), a series of gene-specific and disease-specific interpretation guidelines for further detailed interpretation. The analysis was performed according to the clinical phenotype and family history of the subject, targeting the genes known to be clearly associated with genetic disorders; some genes whose function and pathogenicity are still unknown were excluded from this analysis.

#### Candidate gene qPCR validation

The possible copy number deletion of Xp21.1p11.4 (37431123–38186681) detected was validated by qPCR. Peripheral blood gDNA from family members was extracted using the Blood Genomic DNA Extraction Kit (Tiangen Biotech (Beijing) Co., Ltd.). The design of the primers in exon 5 of the CYBB gene (target region), exon 2 of the XK gene (target region), and intron 5 of the OTC gene (downstream of the target region) is shown in Table 1 of the Supplementary Material. The qPCR reaction system was prepared according to the NovoStart^®^ SYBR qPCR SuperMix Plus kit (Suzhou Coastal Protein Technology Co. Ltd.). The corresponding copy number was calculated using the 2^−ΔΔCT^ method, normalized to the Ct value of the internal reference GAPDH gene, and healthy male subjects were used as controls.

**TABLE 1:** Clinical findings and electroretinogram analysis of a Chinese family with Xp21 DNA microdeletion syndrome.

			Uncorrected		Best-corrected		Scotopic 0.01 ERG		Photopic 3.0 ERG					
			Visual acuity		Visual acuity		b-wave (*µv*)		a-wave (*µv*)		b-wave (*µv*)			
Samples	Age	Onset	OD	OS	OD	OS	OD	OS	OD	OS	OD	OS	Color vision defects	Other signs
1:1	36	None	0.1	0.3	1.0	1.0	Normal	Normal	Normal	Normal	Normal	Normal	Normal	None
1:2	33	None	0.05	0.05	0.4	0.2	Severe decrease	Severe decrease	Severe decrease	Severe decrease	Severe decrease	Severe decrease	Normal	None
II:I	7	2 nd month	0.1	0.12	0.15	0.15	Extinction type	Extinction type	Extinction type	Extinction type	Extinction type	Extinction type	Severe	Horizontal nystagmus

## Results

### General information

The proband, a 7-year-old boy, had complained of progressive night blindness for 4 years. His medical history revealed recurrent lung infections, pulmonary abscesses, and dyspnea since his second month of life, and he was diagnosed with chronic granulomatous disease (CGD) accordingly. At the age of 3, the subject started having blurred vision at night. However, he was not taken to visit the doctor by his parents. Family history revealed that the parents denied consanguineous marriage, and the mother had two pregnancies and two births. The proband’s mother was healthy without any associated non-ocular conditions, such as chronic granulomatous disease. The proband was born at full term by normal delivery and weighed 2,900 g at birth. At the time of examination, he was 7 years old, 115 cm in height, and 19 kg in weight (BMI: 14.4 kg/m2). There was no twitching of the facial or limb muscles, and there were no involuntary movements. The muscle strength of the extremities was normal, and the gait was smooth. He had a lively personality and no hearing, speech, or cognitive impairment in communicating with others. A detailed medical history was obtained and there were no symptoms of cardiovascular disease.

### Ophthalmic examination

**FIGURE 1:**
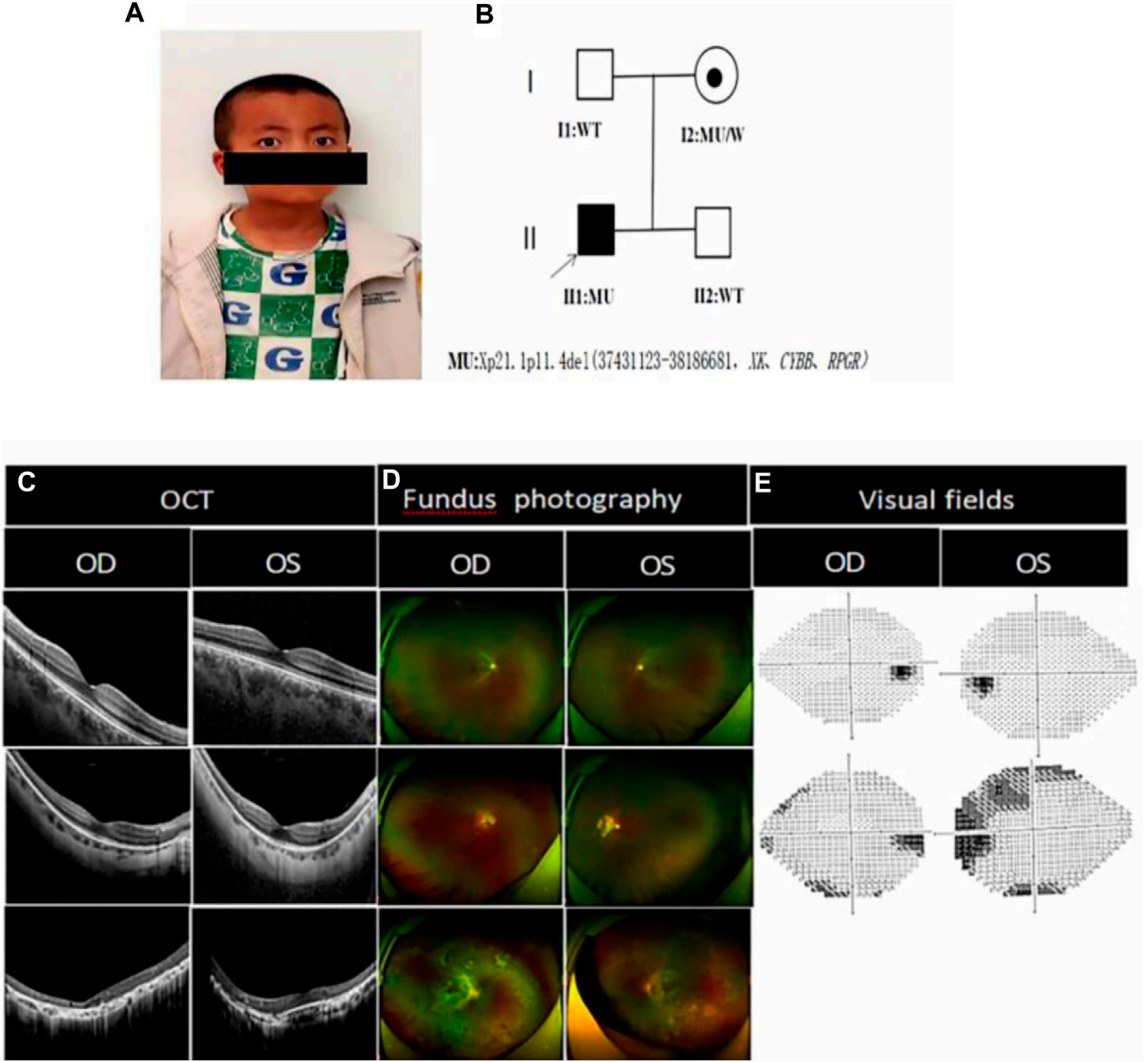
Photographs of the proband’s eye bitmap, the genetic pedigree chart, and a clinical photograph of the proband and his family members: **(A)** Photograph of the proband’s eye bitmap, which shows horizontal nystagmus in both eyes, with fast phase on the left eye and slow phase on the right eye, and a compensatory head posture with the mandible elevated, the face slanted leftward, the eyes slanted rightward, and the angle of cephalic torsion being approximately 20°; **(B)** Genetic pedigree chart of the proband, in which the arrow indicates the proband. **(C)** Optical coherence tomography of I:1, I:2 and II:1. II:1 OCT showed marked thinning of the fovea centralis, structural abnormalities of the chimeric zone, ellipsoid zone and epiretinal membranes, cystic lesion visible in the neuroepithelial layer, granular hyperreflectivity in the RPE layer, RPE breaks, and choroidal thinning; I:2 showed that the fovea centralis became thinner, however, but the fovea centralis remained normal as shown in I:1; **(D)** Fundus photograph of II:1 showed that the retinal optic discs were clear in boundaries and reddish in color, the retinal arteries and veins became thinner, the macular structures were unclear, and multiple osteoblast-like pigmentations were seen at the equator and periphery, accompanied by atrophic pigmentary epithelium. The I:2 retina showed waxy pallor of the optic nerve with unclear boundaries, visible parapapillary chorioretinal atrophy in the periphery of the optic disc, reduced diameter of retinal vessels, and a small amount of osteochromic pigmentation present in the peripheral retina. I:1 fundoscopy was suggestive of normal conditions; **(E)** Visual field: I:2 showed partial loss of peripheral visual field, but the visual field as shown in I:1 was normal.

The best corrected visual acuity (BCVA) was 0.15 (−1.75DC×25°) in the right eye and 0.15 (−1.00DS/-1.75DC×110°) in the left eye (Table 1). IOP:12.3 mmHg in the right eye and 11.6 mmHg in the left eye. No abnormalities were found in the anterior segment of either eye by slit lamp examination in both eyes. An ocular position examination showed horizontal nystagmus in both eyes, with a fast phase in the left eye and a slow phase in the right eye, and a compensatory head posture with the mandible elevated, the face slanted leftward, the eyes slanted rightward, and the angle of cephalic torsion was approximately 20° (Figure 1A). Color vision examination: red-green color blindness in both eyes. Ocular axis: 21.67 mm in the right eye and 21.86 mm in the left eye. OCT showed flattening of the fovea centralis in both eyes, marked retinal thinning, cystic dark areas visible in the neuroepithelial layer, atrophy of the outer structures, granular hyperreflectivity visible in the RPE layer at the corresponding sites, interrupted continuity of the RPE layer, and choroidal thinning (Figure 1C). Fundus photography showed clear borders of the optic discs in both eyes, red color, fine retinal arteries and veins, unclear macular structures, and multiple osteoblast-like pigmentation with pigment epithelial atrophy at the equator and periphery (Figure 1D). Scotopic and photopic ERG responses were absent in both eyes (Table 1). Controlled perimetry: visual field range <10°in both eyes.

The BCVA of the proband’s mother was 0.4 (−13.50 DS/-1.25 DC × 180°) in the right eye and 0.2 (−14.75 DS/-2.50 DC × 171°) in the left eye (Table 1). OCT showed a thinning of the fovea centralis (Figure 1C). A funduscopy showed waxy pallor of the optic nerve with unclear boundaries, visible parapapillary atrophy, the reduced diameter of retinal vessels, and a small amount of osteochromic pigmentation present in the peripheral retina (Figure 1D). As shown in the visual field, peripheral vision was partially lost (Figure 1E). Scotopic and photopic ERG responses were severely reduced in both eyes (Table 1).

The BCVA of the proband’s father was 1.0 (−4.00DS/-1.25DC×3°) in the right eye and 1.0 (−2.00DS/-2.75DC×3°) in the left eye. Fundus photographs, OCT, visual fields (Figure 1), and ERG (Table 1) were normal.

### Blood laboratory tests

**FIGURE 2:**
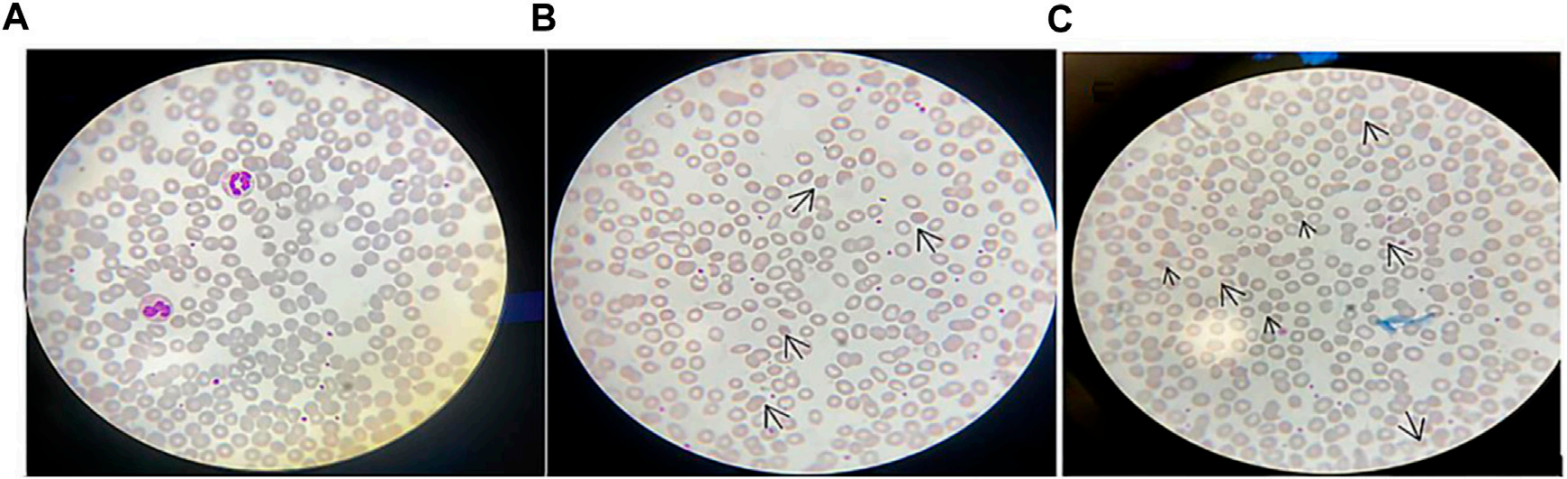
Peripheral blood smear: **(A)** Peripheral blood smear of the proband’s father; **(B)** Peripheral blood smear of the proband’s mother, in which arrows indicate acanthocytes. It can be seen that the acanthocytes were round and had multiple irregular, spaced protrusions of varying length, thickness, and shape, and the proportion of acanthocytes was 1.6%; **(C)** Peripheral blood smear of the proband, in which arrows indicate the proportion of acanthocytes is 3%. Magnification of the protrusions was made microscopically at 100 times.

The subject’s blood laboratory tests showed that leukocytes were 6.13×10^9^/L, erythrocytes were 4.93×10^12^/L, hemoglobin was 130 g/L, and mean hemoglobin concentration was 327 g/L. Liver function, renal function, electrolytes, and blood glucose were normal. Creatine kinase (CK) was 57.00 U/L (normal range: 38–174 U/L). The peripheral blood smear showed 3% acanthocytes in the proband (Figure 2C), 1.6% acanthocytes in the proband’s mother (Figure 2B), and normal in the proband’s father (Figure 2A).

### Genetic test results

**FIGURE 3:**
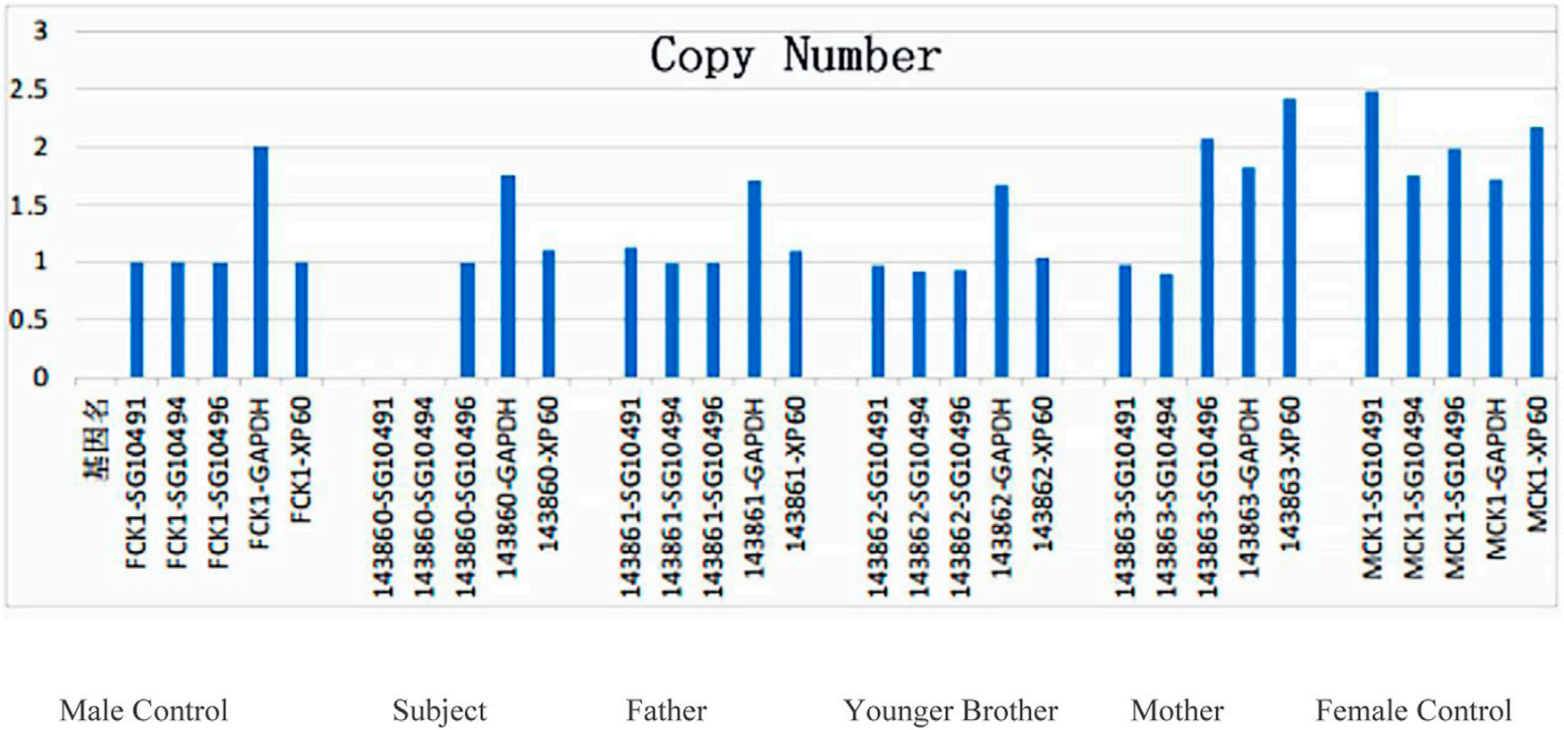
QPCR validation results of the target gene region in the proband’s family: no such mutation was detected in the father and brother, while a heterozygous deletion mutation was not detected in the mother in this region.

WES revealed that the short arm of the X chromosome in the proband had a deletion CNV Xp21.1p11.4 (37431123–38186681) of approximately 0.755 Mb in size and contained such three contiguous OMIM genes as *XK, CYBB,* and *RPGR*. qPCR was performed to verify the possible copy number deletion of Xp21.1p11.4 (37431123–38186681) detected in the subject’s whole-exome sequencing. Two amplicons were designed in the deletion region; one control amplicon was designed downstream of the deletion region; and one control amplicon was designed on each of the autosomes and the X chromosome. qPCR was used to perform relative DNA quantification on the above amplicons and control amplicons (Table 2 in the Supplementary Material). The qPCR results showed that the proband and his mother had the hemizygous deletion variant detected in this region, while the father and younger brother did not have such a variant (Figure 3). The current chromosomal polymorphism database does not cover this region.

**TABLE 2:** Clinical manifestations of Xp21 DNA microdeletion syndrome.

Literature					
	Chronic granulomatous disease	Retinitis pigmentosa	McLeod red cell phenotype	Creatine kinase level	Others
Francke et al. (1985)	+	+	+	High	Duchenne muscular dystrophy
de Saint-Basile et al. (1988)	+	+	+	Normal	Hepatosplenomegaly Moderate anemia
Wilcox et al. (1986)	+	+	+	High	Developmental delay
					Muscle hypotonia
					Large tongue
					Pseudohypertrophy of the calves

### Pathogenicity analysis

The test results showed that the Xp21.1p11.4 segment of the subject might have a hemizygous deletion of the copy number of approximately 0.755 Mb in size. This deletion region contained nine protein-coding genes, including three known OMIM pathogenic genes, namely, *XK*, *CYBB,* and *RPGR*, of which the *CYBB* gene was listed in the ClinGen database as a haploinsufficient gene with a haploinsufficiency (HI) score of 3. Abnormalities in the *CYBB* gene can result in X-linked recessive chronic granulomatous disease (OMIM#306400) or immunodeficiency type 34 (OMIM#300645), and abnormalities in the *RPGR* gene can result in X-linked retinitis pigmentosa type 3 (OMIM#300029), whose clinical phenotypes include reduced central vision and retinitis pigmentosa. According to the ACMG guidelines for CNV interpretation and reporting, deletions in this segment are pathogenic variants.

### Literature review

A review of the reported relevant literature to date has shown that Xp21 DNA microdeletion syndrome has a complex and diverse clinical phenotype (Table 2).

## Discussion

In this study, a copy deletion of approximately 0.755 Mb in size in the short arm of the X chromosome was identified in the proband with RP and CGD, which included complete segmental deletions of three genes, namely, *RPGR*, *CYBB*, and *XK*. Neither the phenotypically normal father nor the sibling brother in the family carried such a deletion variant. The mother carried the same hemizygous deletion in the same region of the X chromosome as the proband, who had a clinical presentation of super-high myopia (−13.50 D OD and −14.75D OS) without RP. The onset of XLRP was earlier than that of autosomal RP, usually in childhood or adolescence, with a mean age of 7.2 ± 1.7 years (Hussels-Maumenee et al., 1975), and patients with XLRP usually presented with a more severe disease. Male subjects carrying the *RPGR* gene variant usually suffered from night blindness early in life, with rapid and severe progressive loss of peripheral vision, followed by progressive loss of central vision in the second to fourth decades life, which could progress to legal blindness (Salvetti et al., 2021; Yang et al., 2021). Clinical features were characterized by bone spicule pigmentation, attenuation of retinal vessels, waxy pallor of the optic nerve, and electroretinogram (ERG) abnormalities (Verbakel et al., 2018). In contrast, female carriers can present with a variety of phenotypes, ranging from asymptomatic, high myopia to severe RP phenotypes (Yang et al., 2021). In this study, the clinical phenotype of the proband’s mother presented super-high myopia (−13.50 D OD and −14.75D OS), and fundus examination suggested high myopic retinopathy in both eyes. It was previously reported that 70% of XLRP was caused by *RPGR* gene variants, and most of them were point variants (Martinez-Fernandez de la Camara et al., 2022). Few patients suffered from XLRP due to a complete segmental deletion of the *RPGR* gene, which has not been reported in China. In a recent article, Mihailovic N et al. reported for the first time a Caucasian male patient who exhibited RP due to a complete segmental deletion of the X-linked RPGR gene, accompanied by a deletion of approximately 378 kb of gene segment Xp11.4 (37814711_38192813) of the Xp11.4 chromosome (Mihailovic et al., 2022). In contrast, in the proband in this study, in addition to the complete segmental deletion of the *RPGR* gene, there was also a complete segmental deletion of the *CYBB* and *XK* genes in the Xp21.1 region. RP typically manifests with night blindness, followed by concentric visual field loss, reflecting the principal dysfunction of rod photoreceptors; central vision loss occurs later in life due to cone dysfunction (Verbakel et al., 2018). Photoreceptor function, as measured by full-field electrophysiological testing, is markedly reduced or even absent (Anasagasti et al., 2012). However, the clinical manifestations of RP are widely variable due to the large number of genes involved, each of which may have several alleles. Systemic symptoms include (rare) hearing loss, sinusitis, asthma, chronic respiratory infections, and even infertility (De Silva et al., 2021). In this study, in addition to the above typical clinical symptoms of RP, the proband also had red-green color blindness and horizontal nystagmus. Systemic clinical manifestations were recurrent pulmonary infections from the second month of life, and pulmonary abscess and respiratory failure could occur when the disease was severe. The diagnosis of chronic granulomatous lesions was confirmed by genetic testing, which confirmed a complete segmental deletion of the *CYBB* gene in the Xp21.1 region. As previously reported, the syndromic RP was also frequent heterogeneous. The most common Usher syndrome is characterized by RP with hearing impairment (Mathur and Yang, 2019). This is followed by the Bardet-Biedl syndrome, which, in addition to RP, includes obesity, polydactyly, hypogonadism, cognitive impairment, and renal abnormalities (Castro-Sánchez et al., 2013; Suspitsin and Imyanitov, 2016). When RP was one of the clinical symptoms, the pathogenic condition involved multiple systems and may have simultaneous or sequential onset, making it difficult for clinicians to find it in time for diagnoses. Therefore, in addition to detailed structural and functional eye examinations, genetic screening should be given high priority in RP patients to identify the pathogenic genes that could contribute to early diagnosis, effective intervention, and long-term follow-up evaluation of these syndromic diseases.

McLeod syndrome (MLS) is an X-linked multisystem disorder caused by *XK* gene variants that clinically manifests with the central nervous system, neuromyopathy, angiocardiopathy, and hematology and may include progressive choreiform movements, cardiomyopathy, peripheral neuropathy, cognitive decline, and psychiatric complications (Jung et al., 2004). The *XK* gene is located in the p21.1 region of the X chromosome and contains three exons with a size of more than 50kb, which is very close to the *CYBB* gene encoding the egp91phox protein in the NADPH oxidative complex enzymes (Wilcox et al., 1986; Supple et al., 2001). The *XK* protein encoded by the *XK* gene has 444 amino acids and 10 transmembrane domains and has similar constitutive properties to transport proteins in the cell membrane, which are found in various tissue systems throughout the body (Lee et al., 2000). Therefore, *XK* gene variants can involve multiple systems throughout the body. The types of *XK* gene variants include nonsense variants, splice site variants, and gene segment deletions (Chen et al., 2014). When *XK* gene segment deletions are excessive, they may involve contiguous *XK* genes, such as the *CYBB* and *RPGR* genes, which could result in “Xp21 DNA microdeletion syndrome”, including XLRP, chronic granulomatous disease, Duchenne muscular dystrophy, and ornithine transcarbamylase deficiency (OTC) (Wilcox et al., 1986; Watkins et al., 2011; Roulis et al., 2018). The serological phenotype of MLS patients is characterized by acanthocytes (Walker et al., 2007), and serum creatine kinase (CK) levels are elevated in the majority of MLS patients (Peikert et al., 2022). The serum CK of the proband in this study was within the normal range, which may be related to the fact that the proband had no clinical manifestations of the muscular system. However, there are also reports that even if the serum CK of patients is found to be elevated, there are no clinical manifestations of the muscular system (Hardie et al., 1991). In this study, a 7-year-old proband with a comprehensive medical history and laboratory tests did not present with hematological, neurological, or motor symptoms related to MLS, which may be related to the fact that the onset of MLS generally occurs between 25 and 61 years of age (Jung et al., 2007). Due to its rarity and the high heterogeneity of its clinical symptoms, MLS is easily confused with extrapyramidal disorders. The *XK* gene variant, a highly specific diagnostic marker for McLeod syndrome, is the “gold standard” for the diagnosis of McLeod syndrome (Ying et al., 2023).

## Conclusion

This paper was the first to report complete segmental deletions of such three contiguous genes as *RPGR, CYBB,* and *XK* in the Xp21 region, causing syndromic XLRP in a Chinese boy with RP and CGD. Our study expands the spectrum of genetic variants and phenotypes of syndromic RP and suggests that genetic testing is not only essential for the early and definitive diagnosis but may also facilitate early intervention and long-term follow-up evaluation of this disease.

## Data Availability

The original contributions presented in the study are included in the article/Supplementary Material, further inquiries can be directed to the corresponding authors.

## References

[B1] Anasagasti A. Irigoyen C. Barandika O. López de Munain A. Ruiz-Ederra J. (2012). Current mutation discovery approaches in Retinitis Pigmentosa. Vis. Res. 75, 117–129. 10.1016/j.visres.2012.09.012 23022136

[B2] Arai T. Zhao M. Kanegane H. van Zelm M. C. Futatani T. Yamada M. (2011). Genetic analysis of contiguous X-chromosome deletion syndrome encompassing the BTK and TIMM8A genes. J. Hum. Genet. 56 (8), 577–582. 10.1038/jhg.2011.61 21753765

[B3] Arnold D. E. Heimall J. R. (2017). A review of chronic granulomatous disease. Adv. Ther. 34 (12), 2543–2557. 10.1007/s12325-017-0636-2 29168144 PMC5709447

[B4] Bruninx R. Lepièce G. L. (2020). Retinitis pigmentosa. Rev. Med. Liege 75 (2), 73–74. 32030928

[B5] Castro-Sánchez S. Álvarez-Satta M. Valverde D. (2013). Bardet-Biedl syndrome: a rare genetic disease. J. Pediatr. Genet. 2 (2), 77–83. 10.3233/PGE-13051 27625843 PMC5020962

[B6] Chen P. Y. Lai S. C. Yang C. C. Lee M. J. Chiu Y. H. (2014). A novel XK gene mutation in a Taiwanese family with McLeod syndrome. J. Neurol. Sci. 340 (1-2), 221–224. 10.1016/j.jns.2014.02.027 24635891

[B7] de Saint-Basile G. Bohler M. C. Fischer A. Cartron J. Dufier J. L. Griscelli C. (1988). Xp21 DNA microdeletion in a patient with chronic granulomatous disease, retinitis pigmentosa, and McLeod phenotype. Hum. Genet. 80 (1), 85–89. 10.1007/BF00451463 3417309

[B8] De Silva S. R. Arno G. Robson A. G. Fakin A. Pontikos N. Mohamed M. D. (2021). The X-linked retinopathies: physiological insights, pathogenic mechanisms, phenotypic features and novel therapies. Prog. Retin Eye Res. 82, 100898. 10.1016/j.preteyeres.2020.100898 32860923

[B9] Fahim A. (2018). Retinitis pigmentosa: recent advances and future directions in diagnosis and management. Curr. Opin. Pediatr. 30 (6), 725–733. 10.1097/MOP.0000000000000690 30234647

[B10] Fahim A. T. Sullivan L. S. Bowne S. J. Jones K. D. Wheaton D. K. H. Khan N. W. (2020). X-chromosome inactivation is a biomarker of clinical severity in female carriers of RPGR-associated X-linked retinitis pigmentosa. Ophthalmol. Retina 4 (5), 510–520. 10.1016/j.oret.2019.11.010 31953110 PMC7211129

[B11] Francke U. Ochs H. D. de Martinville B. Giacalone J. Lindgren V. Distèche C. (1985). Minor Xp21 chromosome deletion in a male associated with expression of Duchenne muscular dystrophy, chronic granulomatous disease, retinitis pigmentosa, and McLeod syndrome. Am. J. Hum. Genet. 37 (2), 250–267. 4039107 PMC1684578

[B12] Hardie R. J. Pullon H. W. Harding A. E. Owen J. S. Pires M. Daniels G. L. (1991). Neuroacanthocytosis. A clinical, haematological and pathological study of 19 cases. Brain 114 (Pt 1A), 13–49. 1998879

[B13] Hosch J. Lorenz B. Stieger K. (2011). RPGR: role in the photoreceptor cilium, human retinal disease, and gene therapy. Ophthalmic Genet. 32 (1), 1–11. 10.3109/13816810.2010.535889 21174525

[B14] Hussels-Maumenee I. Pierce E. R. Bias W. B. Schleutermann D. A. (1975). Linkage studies of typical retinitis pigmentosa and common markers. Am. J. Hum. Genet. 27 (4), 505–508. 17948536 PMC1762794

[B15] Jirapongsananuruk O. Niemela J. E. Malech H. L. Fleisher T. A. (2002). CYBB mutation analysis in X-linked chronic granulomatous disease. Clin. Immunol. 104 (1), 73–76. 10.1006/clim.2002.5230 12139950

[B16] Jung H. H. Danek A. Frey B. M. (2007). McLeod syndrome: a neurohaematological disorder. Vox Sang. 93 (2), 112–121. 10.1111/j.1423-0410.2007.00949.x 17683354

[B17] Jung H. H. Danek A. Walker R. H. Freky D. M. Peikert K. (2004). McLeod neuroacanthocytosis syndrome. [updated 2021 Sep 16]. 20301528

[B18] Justiz-Vaillant A. A. Williams-Persad A. F. Arozarena-Fundora R. Gopaul D. Soodeen S. Asin-Milan O. (2023). Chronic granulomatous disease (CGD): commonly associated pathogens, diagnosis and treatment. Microorganisms 11 (9), 2233. 10.3390/microorganisms11092233 37764077 PMC10534792

[B19] Köker M. Y. Camcıoğlu Y. van Leeuwen K. Kılıç S. Ş. Barlan I. Yılmaz M. (2013). Clinical, functional, and genetic characterization of chronic granulomatous disease in 89 Turkish patients. J. Allergy Clin. Immunol. 132 (5), 1156–1163. 10.1016/j.jaci.2013.05.039 23910690

[B20] Lee S. Russo D. Redman C. M. (2000). The Kell blood group system: kell and XK membrane proteins. Semin. Hematol. 37 (2), 113–121. 10.1016/s0037-1963(00)90036-2 10791880

[B21] Lent-Schochet D. Jialal I. (2023). “Chronic granulomatous disease 2022,” in StatPearls (Treasure Island (FL: StatPearls Publishing). 29630223

[B22] Liu W. Liu S. Li P. Yao K. (2022). Retinitis pigmentosa: progress in molecular Pathology and biotherapeutical strategies. Int. J. Mol. Sci. 23 (9), 4883. 10.3390/ijms23094883 35563274 PMC9101511

[B23] López-Hernández I. Deswarte C. Alcantara-Ortigoza M. Á. Saez-de-Ocariz M. D. M. Yamazaki-Nakashimada M. A. Espinosa-Padilla S. E. (2019). Skewed X-inactivation in a female carrier with X-linked chronic granulomatous disease. Iran. J. Allergy Asthma Immunol. 18 (4), 447–451. 10.18502/ijaai.v18i4.1425 31522453

[B24] Martinez-Fernandez de la Camara C. Cehajic-Kapetanovic J. MacLaren R. E. (2022). Emerging gene therapy products for RPGR-associated X-linked retinitis pigmentosa. Expert Opin. Emerg. Drugs 27 (4), 431–443. 10.1080/14728214.2022.2152003 36562395

[B25] Mathur P. D. Yang J. (2019). Usher syndrome and non-syndromic deafness: functions of different whirlin isoforms in the cochlea, vestibular organs, and retina. Hear Res. 375, 14–24. 10.1016/j.heares.2019.02.007 30831381 PMC6474673

[B26] Mihailovic N. Schimpf-Linzenbold S. Sattler I. Eter N. Heiduschka P. (2022). The first reported case of a deletion of the entire RPGR gene in a family with X-linked retinitis pigmentosa. Ophthalmic Genet. 43 (5), 679–684. 10.1080/13816810.2022.2083181 35652150

[B27] Patnaik S. R. Raghupathy R. K. Zhang X. Mansfield D. Shu X. (2015). The role of RPGR and its interacting proteins in ciliopathies. J. Ophthalmol. 2015, 414781. 10.1155/2015/414781 26124960 PMC4466403

[B28] Peikert K. Hermann A. Danek A. (2022). XK-associated McLeod syndrome: nonhematological manifestations and relation to VPS13A disease. Transfus. Med. Hemother 49 (1), 4–12. 10.1159/000521417 35221863 PMC8832239

[B29] Peng J. Redman C. M. Wu X. Song X. Walker R. H. Westhoff C. M. (2007). Insights into extensive deletions around the XK locus associated with McLeod phenotype and characterization of two novel cases. Gene 392 (1-2), 142–150. 10.1016/j.gene.2006.11.023 17300882 PMC1931494

[B30] Roos D. (2016). Chronic granulomatous disease. Br. Med. Bull. 118 (1), 50–63. 10.1093/bmb/ldw009 26983962 PMC5127417

[B31] Roulis E. Hyland C. Flower R. Gassner C. Jung H. H. Frey B. M. (2018). Molecular basis and clinical overview of McLeod syndrome compared with other neuroacanthocytosis syndromes: a review. JAMA Neurol. 75 (12), 1554–1562. 10.1001/jamaneurol.2018.2166 30128557

[B32] Salvetti A. P. Nanda A. MacLaren R. E. (2021). RPGR-related X-linked retinitis pigmentosa carriers with a severe "male pattern. Ophthalmologica 244 (1), 60–67. 10.1159/000503687 32434206

[B33] Shu X. McDowall E. Brown A. F. Wright A. F. (2008). The human retinitis pigmentosa GTPase regulator gene variant database. Hum. Mutat. 29 (5), 605–608. 10.1002/humu.20733 18361418

[B34] Shu X. Simpson J. R. Hart A. W. Zeng Z. Patnaik S. R. Gautier P. (2012). Functional characterization of the human RPGR proximal promoter. Invest. Ophthalmol. Vis. Sci. 53 (7), 3951–3958. 10.1167/iovs.11-8811 22577079

[B35] Supple S. G. Iland H. J. Barnett M. H. Pollard J. D. (2001). A spontaneous novel XK gene mutation in a patient with McLeod syndrome. Br. J. Haematol. 115 (2), 369–372. 10.1046/j.1365-2141.2001.03121.x 11703337

[B36] Suspitsin E. N. Imyanitov E. N. (2016). Bardet-biedl syndrome. Mol. Syndromol. 7 (2), 62–71. 10.1159/000445491 27385962 PMC4906432

[B37] Tsang S. H. Sharma T. (2018a). Retinitis pigmentosa (Non-syndromic). Adv. Exp. Med. Biol. 1085, 125–130. 10.1007/978-3-319-95046-4_25 30578498

[B38] Tsang S. H. Sharma T. (2018b). X-Linked retinitis pigmentosa. Adv. Exp. Med. Biol. 1085, 31–35. 10.1007/978-3-319-95046-4_8 30578481

[B39] Verbakel S. K. van Huet R. A. C. Boon C. J. F. den Hollander A. I. Collin R. W. J. Klaver C. C. W. (2018). Non-syndromic retinitis pigmentosa. Prog. Retin Eye Res. 66, 157–186. 10.1016/j.preteyeres.2018.03.005 29597005

[B40] Walker R. H. Danek A. Uttner I. Offner R. Reid M. Lee S. (2007). McLeod phenotype without the McLeod syndrome. Transfusion 47 (2), 299–305. 10.1111/j.1537-2995.2007.01106.x 17302777

[B41] Watkins C. E. Litchfield J. Song E. Jaishankar G. B. Misra N. Holla N. (2011). Chronic granulomatous disease, the McLeod phenotype and the contiguous gene deletion syndrome-a review. Clin. Mol. Allergy 9, 13. 10.1186/1476-7961-9-13 22111908 PMC3267648

[B42] Wilcox D. E. Cooke A. Colgan J. Boyd E. Aitken D. A. Sinclair L. (1986). Duchenne muscular dystrophy due to familial Xp21 deletion detectable by DNA analysis and flow cytometry. Hum. Genet. 73 (2), 175–180. 10.1007/BF00291610 3721503

[B43] Yang J. Zhou L. Ouyang J. Xiao X. Sun W. Li S. (2021). Genotype-phenotype analysis of RPGR variations: reporting of 62 Chinese families and a literature review. Front. Genet. 12, 600210. 10.3389/fgene.2021.600210 34745198 PMC8565807

[B44] Ying Y. Yu S. Zhang J. He J. Xu X. Hong X. (2023). A case of McLeod syndrome caused by a nonsense variation c.942G>A in the XK gene: a case report. Front. Genet. 14, 1073139. 10.3389/fgene.2023.1073139 36816020 PMC9929429

