# Supplementary material for: Xp21 DNA microdeletion syndrome in a Chinese family: clinical features show retinitis pigmentosa and chronic granuloma
Source: Front Genet. 2024 Jan 26;14:1276227. doi: 10.3389/fgene.2023.1276227 (PMC10853329; doi:10.3389/fgene.2023.1276227)
Supplement: Supplementary file 1 [file Table1.DOCX]

| Raw sequencing data volume（Mb） | 12,649.75 |
| --- | --- |
| Total number of sequencing reads (entries) | 84,331,636 |
| Average sequencing depth in the target region（X） | 136.08 |
| Average sequencing depth of target region ≥ 1X coverage (%) | 99.75 |
| Average sequencing depth of target region ≥ 10X coverage (%) | 99.39 |
| Average sequencing depth of target region ≥ 30X coverage (%) | 98.56 |
| 20% X average depth coverage (%) | 98.99 |
| Average sequencing depth of mitochondrial genome (X) | 6,302.85 |
| Q30 passing rate (%) | 93.29 |

TABLE 2 Location for target gene amplicon location

| Amplicon | Genomic Location（hg19） | Gene Name | Exon/Intron Location | Remark |
| --- | --- | --- | --- | --- |
| SG10491 | chrX:37652707-37653291 | CYBB | exon5 | Target region  chrX:37431123-38186681 |
| SG10494 | chrX:37553554-37553663 | XK | exon2 | Target region  chrX:37431123-38186681 |
| SG10496 | chrX:38261023-38261119 | OTC | Intron5 | Downstream of target region |
| GAPGH | / | / | / | Autosomal control |
| XP60 | / | / | / | X chromosome control |

TABLE 1 DNA sequencing parameters of the child patient and family members
